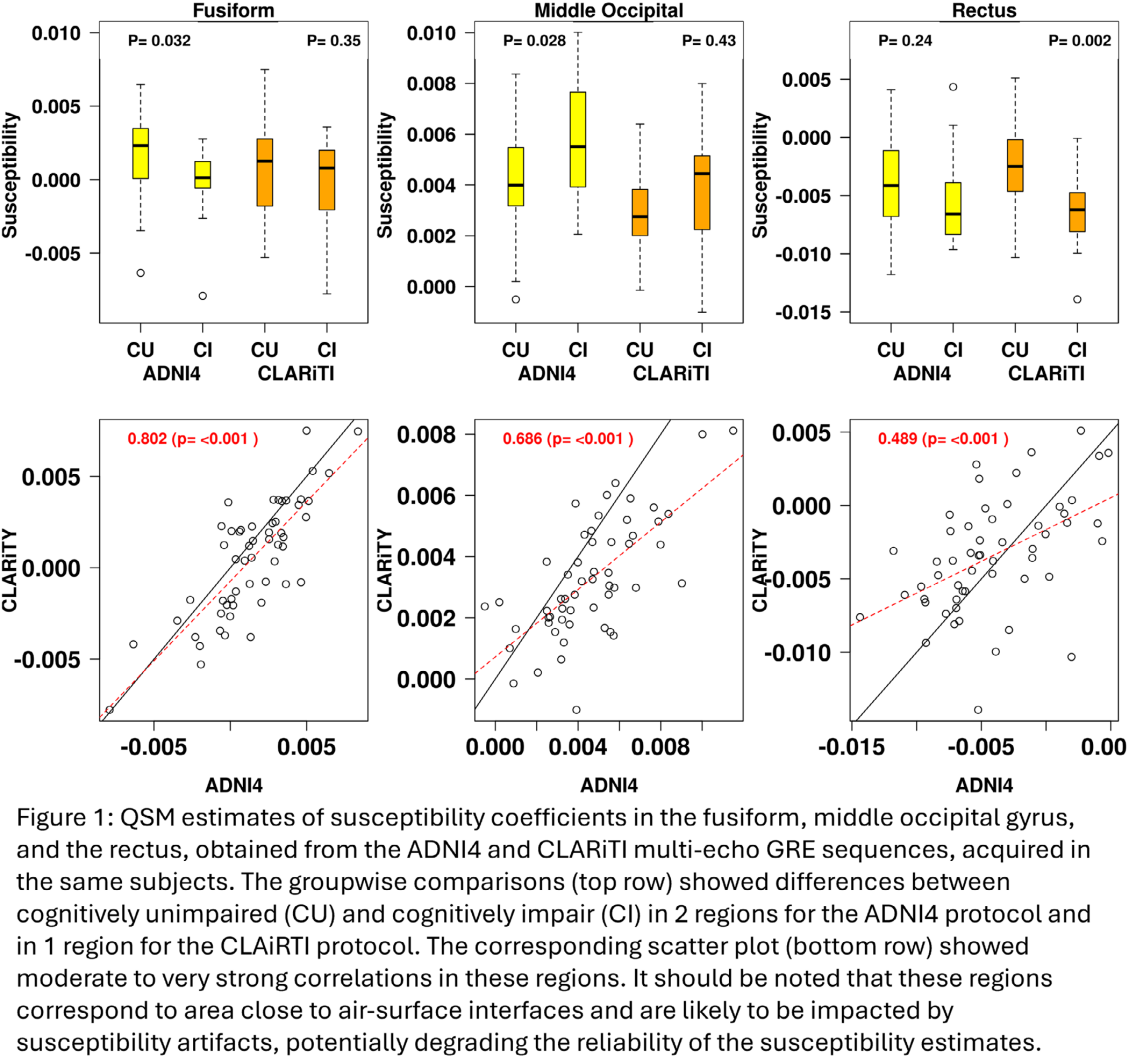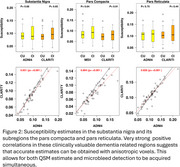# Determining the Impact of Voxel Size on the Clinical Utility of Quantitative Susceptibility Imaging in Multicenter Clinical Trials

**DOI:** 10.1002/alz70862_109957

**Published:** 2025-12-23

**Authors:** Arvin Arani, Scott A. Przybelski, Bret J. Borowski, Matthew L. Senjem, Petrice M Cogswell, Prashanthi Vemuri, Kejal Kantarci, Clifford R. Jack

**Affiliations:** ^1^ Mayo Clinic, Rochester, MN USA; ^2^ Department of Quantitative Health Sciences, Mayo Clinic, Rochester, MN USA; ^3^ Department of Radiology, Mayo Clinic, Rochester, MN USA

## Abstract

**Background:**

T2*‐weighted imaging is of paramount importance for patient treatment eligibility and safety monitoring in anti‐amyloid pharmaceutical clinical trials and clinical care. Recently with the adoption of multi‐echo GRE (GRE) sequences, susceptibility weighted imaging (SWI), and quantitative susceptibility mapping (QSM) can be obtained in the same acquisition. In 2024, the QSM Consensus Organization Committee released a white paper recommending at least 1 mm isotropic voxel size for QSM. In clinical workflows microbleed detection is performed with higher in‐plane resolution (0.5x0.5mm^2^), and large out‐of‐plane resolution (∼1.8mm), which was used in the ADNI4 protocol. In the CLARiTI protocol, a 1mm isotropic resolution was adopted. The objective of this work was to compare susceptibility estimates between the ADNI4 (0.5x0.5x1.8mm^3^) and CLARiTI (1mm^3^) protocols.

**Method:**

The ADNI4 and CLARiTI QSM protocols were acquired on forty‐seven subjects (33 cognitively unimpaired (CU), 14 cognitively impaired (CI), in the same scan session. The groups were defined by their Clinical Demential Rating (CDR) global score (CDR global = 0 for CU, and CDR global >= 0.5 for CI subjects). QSM images were generated using publically available software (STI Suite, UC Berkeley). Mean susceptibility values in atlas regions were compared.

**Result:**

Significant group differences (CU vs. CI) were observed in the fusiform and middle occipital gyrus with the ADNI4 protocol, only in the rectus with the CLARiTI protocol (Figure 1). These three regions are small in size and closer to surface‐air interfaces, which may have impacted estimation accuracy. However, in the substantia nigra and its sub‐regions (pars compacta and pars reticulata), which shows increased susceptibility in Lewy body disease, the ADNI4 and CLARiTI protocols were in good agreement (r^2^ > 0.9) (Figure 2).

**Conclusion:**

Group differences were observed between the ADNI4 and CLARiTI QSM only in 3 small regions close to surface‐air interfaces in this small dataset. The two techniques showed highly correlated (r^2^ = 0.93) estimates in the substantia nigra, a region with high susceptibility due to iron deposition. Strong correlations between the two protocols are encouraging. Radiologists have historically preferred submillimeter in‐plane resolution for microbleed detection, which is more time manageable with anisotropic imaging.